# Effect of Zr Additions on the Microstructure and Elevated-Temperature Mechanical Properties of Al–Cu–Mg–Ag–Zn–Mn–Zr Alloys

**DOI:** 10.3390/ma18174062

**Published:** 2025-08-29

**Authors:** Haoyang Fu, Hongda Yan, Bin Wei, Bin Sun, Zihang Liu, Weihong Gao

**Affiliations:** 1 College of Materials Science and Chemical Engineering, Harbin Engineering University, Harbin 150001, China; 18943369175@163.com (H.F.); yanhongda8621@163.com (H.Y.); sunbin2040@163.com (B.S.); 2 Wuxi Little Swan Company Limited, Wuxi 214028, China; weibin1997917@163.com; 3 State Key Laboratory of Precision Welding & Joining of Materials and Structures, Harbin Institute of Technology, Harbin 150001, China; zihangliu@hit.edu.cn; 4 Yangtze Delta Region Advanced Research Institute, Harbin Engineering University, Nantong 226000, China

**Keywords:** Al–Cu–Mg–Ag alloy, zirconium additions, microstructure, elevated-temperature mechanical properties, heterogeneous nucleation

## Abstract

This study systematically investigates the influence of Zr additions (0–0.24 wt.%) on the microstructure evolution and mechanical properties of Al–4.0Cu–0.5Mg–0.5Zn–0.5Mn–0.4Ag alloys under peak-aged conditions. Alloys were subjected to homogenization (420 °C/8 h + 510 °C/16 h), solution treatment (510 °C/1.5 h), and aging (190 °C/3 h). Microstructural characterization via OM, SEM, EBSD, and TEM revealed that Zr refines grains and enhances recrystallization resistance through coherent Al_3_Zr precipitates, which pin grain boundaries and dislocations. However, excessive Zr (0.24 wt.%) induces heterogeneous grain size distribution and significant Schmid factor variations, promoting stress concentration and premature intergranular cracking. Crucially, Al_3_Zr particles act as heterogeneous nucleation sites for Ω-phase precipitates, accelerating their nucleation near grain boundaries, refining precipitates, and narrowing precipitate-free zones (PFZs). Mechanical testing demonstrated that the Al–4.0Cu–0.5Mg–0.5Zn–0.5Mn–0.4Ag alloy exhibits optimal properties: peak tensile strength of 368.8 MPa and 79.8% tensile strength retention at 200 °C. These improvements are attributed to synergistic microstructural modifications driven by controlled Zr addition, establishing Al–4.0Cu–0.5Mg–0.5Zn–0.5Mn–0.4Ag–0.16Zr as a promising candidate for high-temperature aerospace applications.

## 1. Introduction

Al–Cu–Mg alloys exhibit low density, good corrosion resistance, excellent plasticity, and favorable room-temperature strength [1,2]. Compared to titanium alloys and composite materials, this alloy system offers significant cost advantages. These alloys show promise in meeting the economic and heat resistance requirements for supersonic aircraft, representing a highly prospective development direction as candidate materials. However, with advancements in aerospace technology, modern supersonic aircraft can achieve cruise speeds of Mach 2.4 or higher, where the skin temperature can reach or exceed 190 °C. If the skin, made from 2xxx series aluminum alloys, is exposed long-term to environments above 150 °C, the precipitates within the alloy undergo coarsening or phase transformation, leading to the degradation of the microstructure and potential failure, which may cause accidents [3].

**Figure 1:**
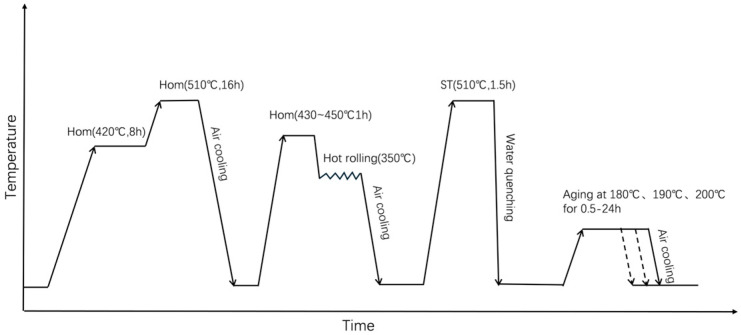
Schematic diagram of the heat treatment procedure.

Alcoa in the United States pioneered the development of the Ag–containing KO–1 alloy and achieved its industrial production. The addition of Ag to Al–Cu–Mg alloys causes Mg and Ag to co-segregate, forming atomic clusters known as Mg–Ag co-clusters. These serve as nucleation sites for the Ω-phase [4,5,6], promoting its precipitation. Consequently, the Ω-phase becomes the primary strengthening phase while suppressing the precipitation of θ’- and S’-phases, thereby altering the precipitation sequence of secondary phases during the aging process of Al–Cu–Mg alloys [4]. Zhou et al. [7] investigated the effect of varying Ag content on the nucleation and thermal stability of the Ω-phase. They found that, as the Ag content increased from 0.14 wt.% to 0.57 wt.%, the number density of the Ω-phase increased dramatically, leading to significant improvements in peak-aged hardness and mechanical properties, as shown in Figure 1 Furthermore, the addition of Ag inhibits the nucleation of the θ’-phase by suppressing the formation of defects like dislocations, thereby enhancing the homogeneous nucleation tendency of the Ω-phase. Increasing Ag content also increases the resistance to thickening of the Ω-phase and improves its thermal stability [8,9,10,11].

The microstructure and properties of the alloy can also be enhanced by adding minor amounts of transition elements (such as Mn, Zr) and rare earth elements. Mn exerts a significant strengthening effect on Al–Cu–Mg alloys and improves their high-temperature stability. Adding an appropriate amount of Mn can suppress the precipitation of brittle phases associated with impurity elements like Fe [12]. Additionally, during homogenization, Mn can form a high-temperature strengthening phase called the T phase with Al and Cu [13]. As a dispersed phase, the T phase impedes grain boundary movement, inhibits recrystallization, hinders the growth of recrystallized grains, and thus controls grain size [14,15]. However, excessive Mn addition can lead to the coarsening of Mn–containing phases, significantly impairing the alloy’s plasticity [16,17].

The primary role of Zr addition is to refine grains and increase the alloy’s recrystallization resistance, thereby enhancing its high-temperature creep resistance and thermal stability [18,19]. This occurs because, during the homogenization treatment of Zr–containing aluminum alloys, Al_3_Zr particles precipitate at grain or sub-grain boundaries. These Al_3_Zr particles, which are coherent with the matrix, strongly impede dislocation movement and grain boundary migration, increasing the alloy’s resistance to recrystallization [20]. Consequently, recrystallization during subsequent heat treatments is suppressed [21,22], preserving the deformed microstructure, reducing dislocation pile–ups, alleviating stress concentrations, and ultimately improving the alloy’s strength and toughness [23,24,25,26]. Currently, systematic studies on the influence of Zr addition on the microstructure and mechanical properties of Al–Cu–Mg–Ag alloys are lacking. This paper investigates the effect of varying Zr content on the properties of Al–Cu–Mg–Zn–Mn–Ag–Zr alloy by observing microstructural evolution and conducting performance tests, aiming to identify the optimal composition for Al–Cu–Mg–Zn–Mn–Ag–Zr alloy.

## 2. Materials and Methods

Based on the ideal composition of the alloys Al–4.0Cu–0.5Mg–0.4Ag–(x = 0, 0.08, 0.16 and 0.24) Zr (in wt.%), alloys for the experiment were melted and cast at Northeast Light Alloy Company, Harbin, China. The alloys were prepared through direct chilling by pure aluminum ingot (99.99% pure), magnesium ingot (99.9% pure), zinc ingot (99.9% pure), Al–40wt%Cu, Al–10wt%Mn, Al–10wt%Ag and Al–10wt%Zr master alloys in air. The alloy compositions were measured using an Inductively Coupled Plasma-Atomic Emission Spectrometry (ICP-AES). Three samples were taken from the upper, middle, and lower parts of ingots with different compositions, and the average value was calculated after testing each sample three times. Their actual compositions are shown in Table 1. The ingot dimensions were 5 cm × 10 cm × 30 cm. After casting, the ingots underwent homogenization treatment and hot rolling. The hot rolling process involved holding at 430–460 °C, with the final rolling temperature controlled at 350 °C and a reduction rate of 60%. The rolling was completed in five passes.

These specimens were solid-solution treated at 510 °C for 1.5 h, subsequently water quenched, and then immediately aged at 190 °C for 3 h. The heat treatment procedure has been shown in Figure 1. Mechanical properties at room and elevated temperatures were determined using an AG-Xplus electronic universal testing machine (Shimadzu, Kyoto, Japan) following the GBT 228.1-2010 standard [27] at a tensile velocity of 2 mm/min. Three specimens per heat treatment condition were tested, and the results were averaged.

Microstructural characterization employed several techniques, like optical microscopy (OM) and scanning electron microscopy (SEM) using an FEI APREO S LOVAC instrument (Thermo Fisher Scientific, Hillsboro, OR, USA) equipped with a backscattered electron detector. Electron backscatter diffraction (EBSD) performed on the same FEI APREO S LOVAC SEM fitted with a TSL (Tex Sem Lab) data acquisition system. SEM/EBSD used 20 kV voltage and 0.2 μm step size. Data analysis used EDAX Tex-SEM TSL-OIM software (version 7.1), with results presented as inverse pole figure (IPF) maps illustrating crystallographic orientations relative to the sample direction. Transmission electron microscopy (TEM) was conducted on an FEI TECNAI G220. Sample preparation involved twinjet electropolishing at −30 °C using a solution of 75% methanol and 25% nitric acid.

## 3. Results and Discussion

**Figure 2:**
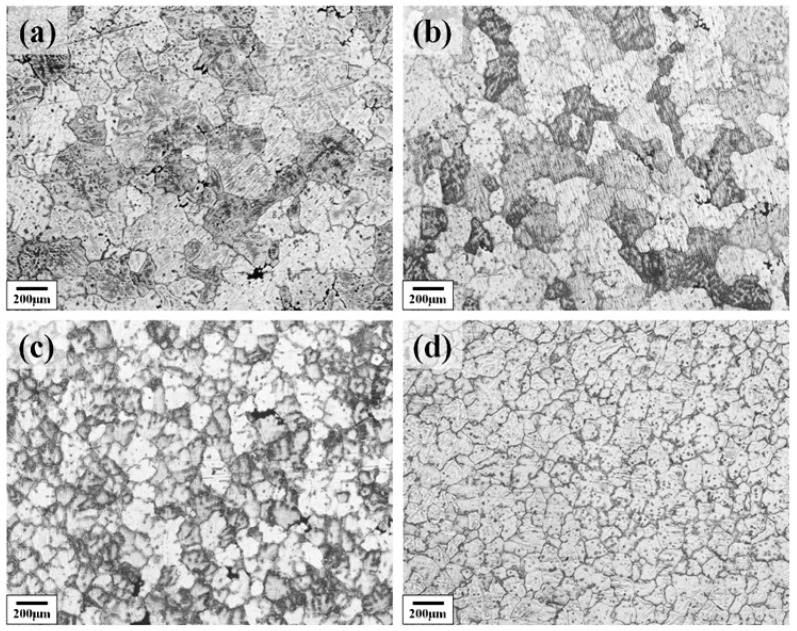
The as-cast metallographic microstructure of the Al–4.0Cu–0.5Mg–0.5Zn–0.5Mn–0.4Ag–xZr (in wt.%) alloys. (**a**) x = 0; (**b**) x = 0.07; (**c**) x = 0.16; (**d**) x = 0.24.

Figure 2 shows the as-cast metallographic microstructure of the Al–4.0Cu–0.5Mg–0.5Zn–0.5Mn–0.4Ag–(x = 0, 0.08, 0.16, and 0.24)Zr (in wt.%) alloys. It can be seen from the images that the grains of the as-cast alloy are gradually refined with increasing Zr content in the alloy. The grain size gradually reduced from about 200 μm to 70 μm. This pronounced initial refinement is attributed to the potent grain-refining efficacy of Zr solute, operating through a growth restriction factor (GRF) mechanism.

**Figure 3:**
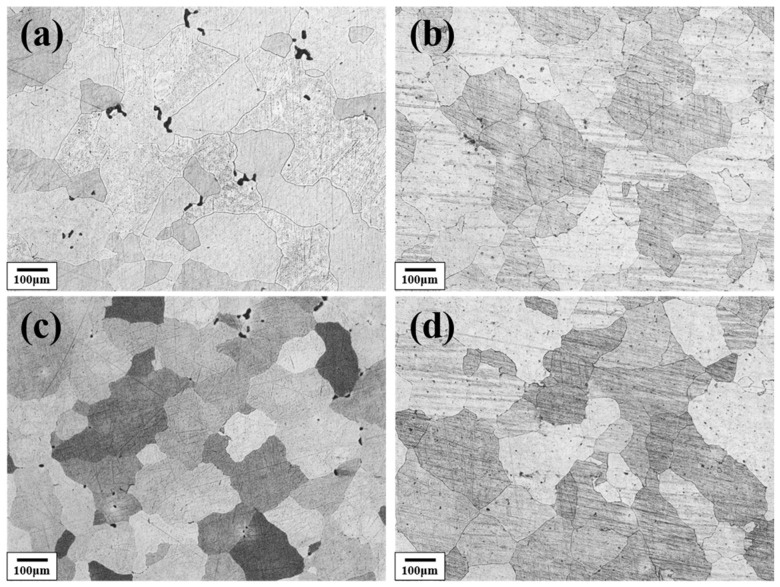
The metallographic microstructure of the Al–4.0Cu–0.5Mg–0.5Zn–0.5Mn–0.4Ag–xZr (in wt.%) alloys after 420 °C/8 h + 510 °C/16 h homogenization treatment. (**a**) x = 0; (**b**) x = 0.07; (**c**) x = 0.16; (**d**) x = 0.24.

Figure 3 shows the solution-treated microstructure of the Al–4.0Cu–0.5Mg–0.5Zn–0.5Mn–0.4Ag–(x = 0, 0.08, 0.16, and 0.24)Zr (in wt.%) alloys after homogenization treatment at 420 °C/8 h + 510 °C/16 h. It can be observed that the alloys containing 0.08 wt.%, 0.15 wt.%, and 0.24 wt.% Zr retained relatively fine grains, even after this prolonged high-temperature treatment. In contrast, the Zr–free alloy exhibited a pronounced coarsening tendency and the grain size is about three times compared to the other three alloys.

**Figure 4:**
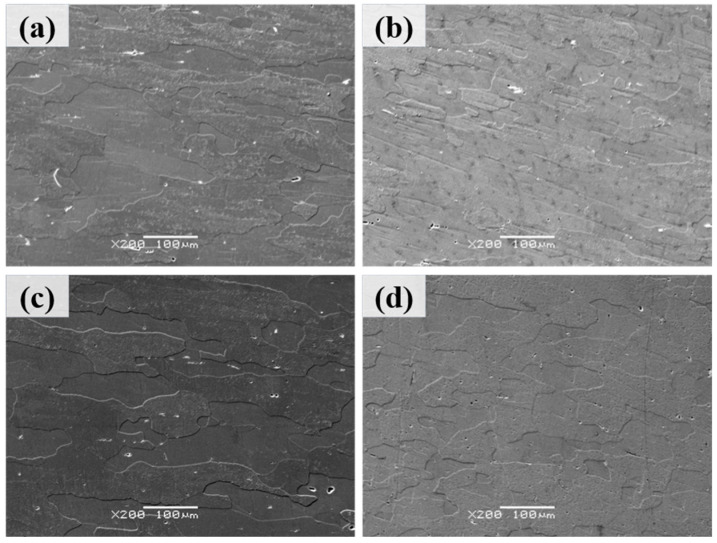
The backscattered electron (BSE) micrographs of Al–4.0Cu–0.5Mg–0.5Zn–0.5Mn–0.4Ag–xZr (in wt.%) alloys after solution treatment at 510 °C/1.5 h. (**a**) x = 0, (**b**) x = 0.08, (**c**) x = 0.16, and (**d**) x = 0.24.

Figure 4 presents the backscattered electron (BSE) micrographs of Al–4.0Cu–0.5Mg–0.5Zn–0.5Mn–0.4Ag–(x = 0, 0.08, 0.16, and 0.24)Zr (in wt.%) alloys after solution treatment at 510 °C/1.5 h. It can be observed that the three Zr–containing alloys partially retained their elongated grain structure from hot rolling. In contrast, the Al–4.0Cu–0.5Mg–0.5Zn–0.5Mn–0.4Ag alloy (without Zr) exhibited more pronounced grain growth after the 510 °C/1.5 h heat treatment. These results can once again demonstrate that the addition of zirconium (Zr) enhances the recrystallization resistance of the alloy.

**Figure 5:**
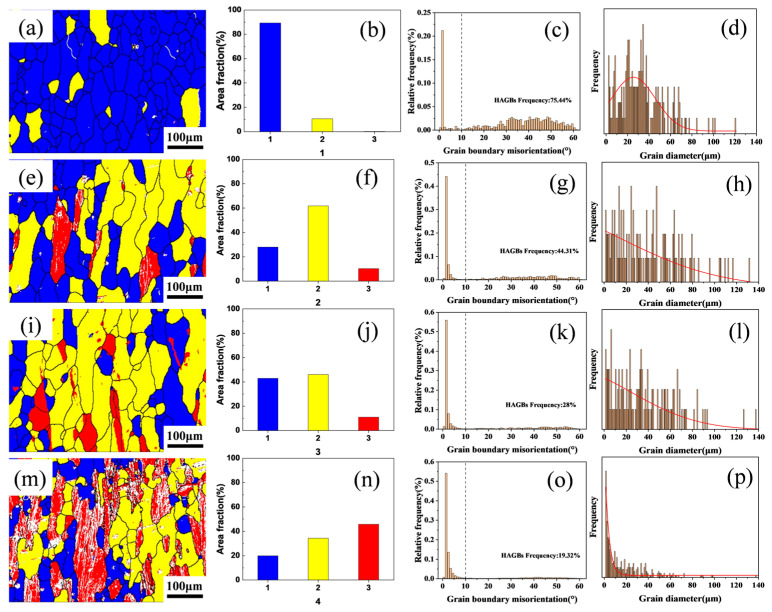
The electron backscatter diffraction (EBSD) micrographs of Al–4.0Cu–0.5Mg–0.5Zn–0.5Mn–0.4Ag–(x = 0, 0.08, 0.16, and 0.24)Zr (in wt.%) alloys after solution treatment. (**a**–**d**) x = 0, (**b**–**h**) x = 0.08, (**i**–**l**) x = 0.16, and (**m**–**p**) x = 0.24.

To further investigate the effect of varying Zr content on the recrystallization resistance of the alloys, electron backscatter diffraction (EBSD) analysis was performed on the solution-treated microstructures of Al–4.0Cu–0.5Mg–0.5Zn–0.5Mn–0.4Ag–xZr (in wt.%) alloys. The results are presented in Figure 5. In the EBSD maps, recrystallized grains are colored blue, sub-grains are colored yellow, and deformed grains are colored red. Regarding grain boundaries, boundaries with misorientation angles θ > 10° are defined as High-Angle Boundaries (HABs) and are represented by black lines, while boundaries with misorientation angles 2° < θ < 10° are defined as Low-Angle Boundaries (LABs) and are represented by white lines.

As evidenced in Figure 5a,e,l,m, variations in Zr content exert a significant influence on grain size and morphology. The Al–4.0Cu–0.5Mg–0.5Zn–0.5Mn–0.4Ag alloy exhibits extensive recrystallized regions, demonstrating a recrystallization fraction of 89.29% and a high-angle boundary (HAB) proportion of 75.44%, indicating predominantly equiaxed recrystallized grains, as shown in Figure 5b,f,j,n. In the Al–4.0Cu–0.5Mg–0.5Zn–0.5Mn–0.4Ag–0.08Zr alloy, recrystallized areas decrease while sub-grain and deformed grain fractions increase, accompanied by a reduced HAB frequency of 44.31%. This composition features elongated grains with markedly diminished equiaxed recrystallized structures. Al–4.0Cu–0.5Mg–0.5Zn–0.5Mn–0.4Ag–0.16Zr alloy shows a recrystallization fraction of 27.95%, containing distinctly elongated grains aligned with the rolling direction and exhibiting relatively uniform grain size. With further Zr addition, the Al–4.0Cu–0.5Mg–0.5Zn–0.5Mn–0.4Ag–0.24Zr alloy registers a recrystallization fraction of 19.91% and sub-grain fraction of 34.32%, retaining substantial deformed grains. These red-colored deformed grains contain abundant Low-Angle Boundaries (LABs), display significant grain size heterogeneity, and exhibit a markedly reduced HAB proportion of only 19.32%. Complementary quantitative analyses of HAB/LAB distributions and grain diameter statistics across Al–4.0Cu–0.5Mg–0.5Zn–0.5Mn–0.4Ag–xZr (in wt.%) alloys are presented in Figure 5c,g,k,o and Figure 5d,h,l,p, respectively.

Figure 5d,h,l,p displays the grain diameter distributions across the Al–4.0Cu–0.5Mg–0.4Ag–xZr (in wt.%) alloys. With increasing Zr content, the probability of finer grains rises significantly. However, excessive Zr addition (0.24 wt.%) leads to heterogeneous grain size distribution, as evidenced in Figure 5n, where substantial grain size variations are observed. This heterogeneity arises because high Zr concentrations promote the formation of coarse primary Al_3_Zr particles, thereby disrupting uniform grain growth.

Collectively, these analyses demonstrate that elevated Zr content enhances the alloy’s recrystallization resistance and preserves a greater fraction of the deformation microstructure inherited from hot rolling. Nevertheless, excessively high Zr levels (≥0.24 wt.%) induce non-uniform grain size distributions, which may detrimentally affect mechanical properties.

**Figure 6:**
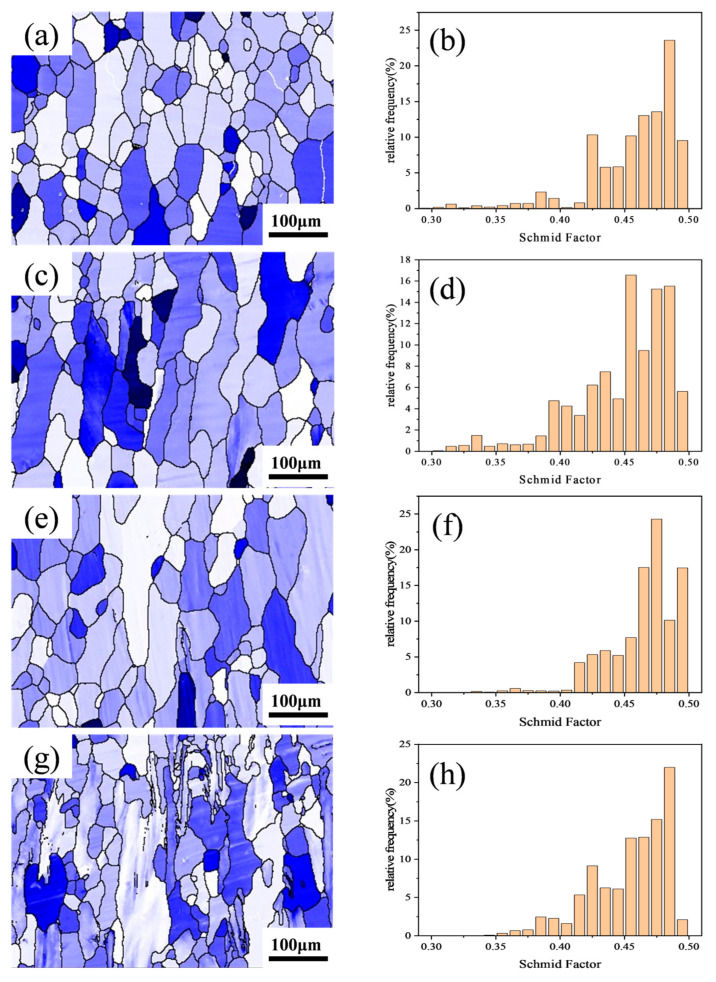
Schmid factor distribution maps and statistical charts for the Al–4.0Cu–0.5Mg–0.5Zn–0.5Mn–0.4Ag–xZr (in wt.%) alloys. (**a**,**e**) x = 0, (**b**,**f**) x = 0.08, (**c**,**g**) x = 0.16, and (**d**,**h**) x = 0.24.

Figure 6 shows the {111} <11$\overset{-}{1}$> Schmid factor distribution maps and statistical charts for the Al–4.0Cu–0.5Mg–0.5Zn–0.5Mn–0.4Ag–xZr (in wt.%) alloys. The Schmid factor values in the figures range from 0.3 to 0.5. A darker grain color indicates a lower Schmid factor, signifying greater difficulty in grain deformation. Larger differences in Schmid factor values between adjacent grains increase the likelihood of crack formation at grain boundaries.

As observed in Figure 6, newly recrystallized grains typically exhibit higher Schmid factor values compared to deformed structures, meaning that they deform more readily. A comparison of Figure 6e–h reveals that the Al–4.0Cu–0.5Mg–0.5Zn–0.5Mn–0.4Ag alloy contains a small number of sub-grain regions (black) with relatively low Schmid factor values, but the Al–4.0Cu–0.5Mg–0.5Zn–0.5Mn–0.4Ag–0.08Zr alloy retains some deformed structures (dark blue). In the Al–4.0Cu–0.5Mg–0.4Ag–0.16Zr alloy, the overall color becomes lighter. The deformed structures within it still maintain lower Schmid factor values, resulting in a smaller overall difference in Schmid factor values. In the Al–4.0Cu–0.5Mg–0.5Zn–0.5Mn–0.4Ag–0.24Zr alloy, a significant number of deformed structures appear pale–colored, almost all exhibiting higher Schmid factor levels. Coarse deformed grains remain prevalent. Conversely, the recrystallized grains and sub-grain structures within this alloy possess lower Schmid factor values. Stress concentration tends to occur near the coarse deformed grains with higher Schmid factors. Consequently, dislocations continuously accumulate in these regions, leading to the initiation of intergranular cracks and thereby impairing the alloy’s performance.

**Figure 7:**
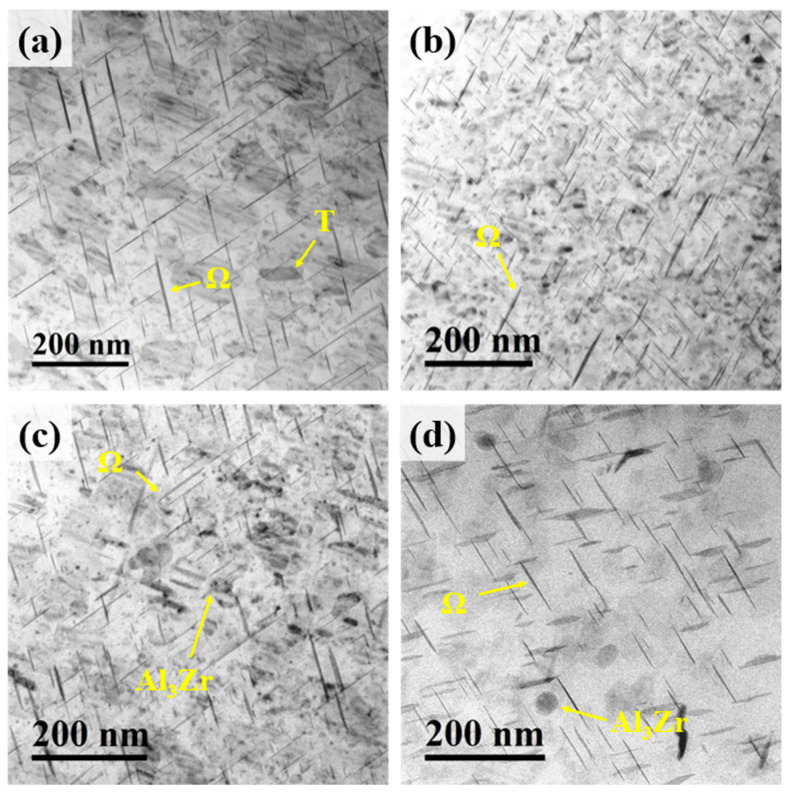
TEM images of the peak-aged Al–4.0Cu–0.5Mg–0.5Zn–0.5Mn–0.4Ag–xZr (in wt.%) alloys. (**a**) x = 0; (**b**) x = 0.08; (**c**) x = 0.16; (**d**) x= 0.24.

Figure 7 shows the TEM images of the peak-aged Al–4.0Cu–0.5Mg–0.5Zn–0.5Mn–0.4Ag–(x = 0, 0.08, 0.16, and 0.24)Zr (in wt.%) alloys. A high density of needle-/plate-like precipitates and a small number of rod-shaped precipitates are distributed within the matrix of all alloys. Additionally, spherical precipitates are observed in the Al–4.0Cu–0.5Mg–0.5Zn–0.5Mn–0.4Ag–0.16Zr and Al–4.0Cu–0.5Mg–0.5Zn–0.5Mn–0.4Ag–0.24Zr alloys. After the two–step homogenization treatment, the Al_3_Zr phase becomes finer, more uniformly distributed, and more dispersed. Moreover, the spherical precipitates in the former condition are found to be more numerous and smaller in size compared with the latter [28]. The needle-/plate-like precipitates are identified as the Ω-phase, the rod-shaped precipitates as the θ’-phase, and the spherical precipitates as the Al_3_Zr phase. No S-phase precipitates were detected in these alloys, or their content is extremely low. This indicates that reducing the Mg content effectively suppresses the precipitation of the S-phase.

**Figure 8:**
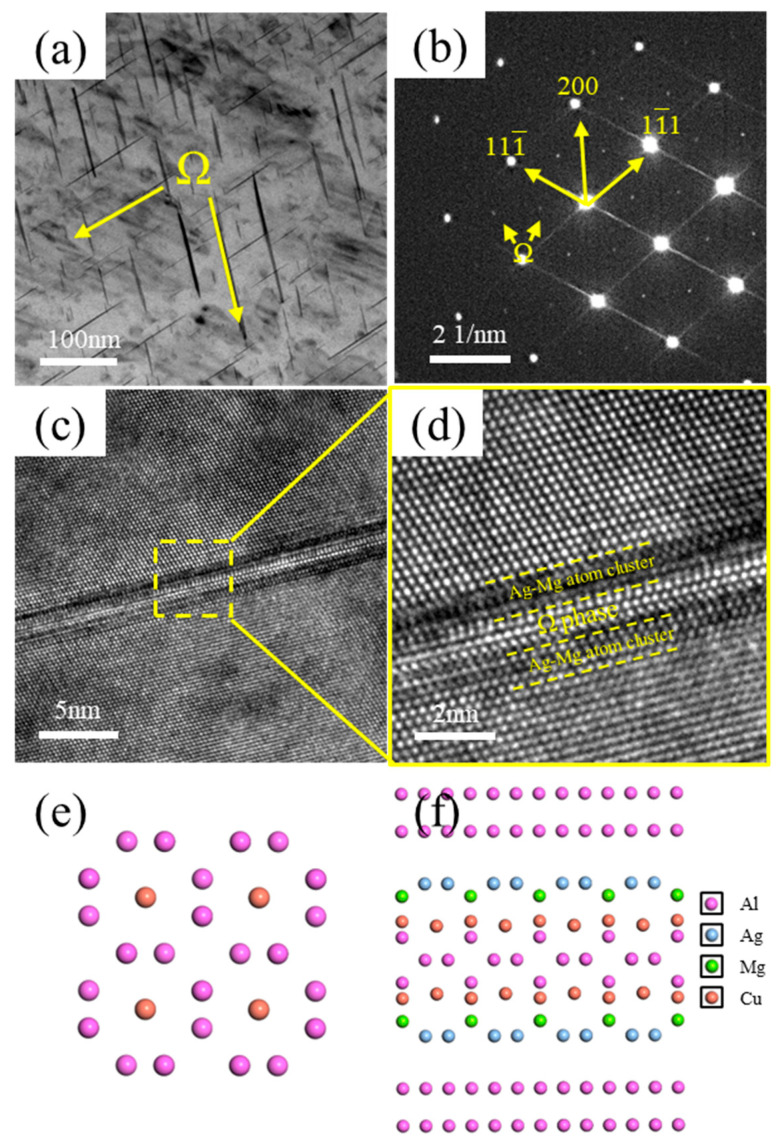
TEM images of the Ω-phase. (**a**) Bright–field (BF) image of Ω-phase; (**b**) Selected area electron diffraction (SAED) pattern corresponding to (**a**), incident beam direction // <011>α; (**c**) High-resolution TEM (HRTEM) image of Ω-phase; (**d**) Enlarged view of the boxed region in (**c**); (**e**) Structure of Ω-phase; (**f**) Interface model between Ω-phase and Al matrix.

Figure 8 presents TEM images of the Ω-phase in the alloy. As shown in Figure 8a, the Ω-phase exhibits a plate-like morphology and is distributed within the alloy matrix. Its corresponding diffraction pattern is shown in Figure 8b. Figure 8c shows a high-resolution TEM (HRTEM) image of the Ω-phase, and Figure 8d displays its corresponding Fast Fourier Transform (FFT) pattern. The interfacial structure between the Ω-phase, the Ag–Mg bilayer, and the Al matrix is depicted in Figure 8f, while the structure of the Ω-phase itself is illustrated in Figure 8e. It can be observed that distinct Ag–Mg bilayers flank both sides of the Ω-phase, effectively sandwiching it. Due to the blocking effect of the Ag–Mg bilayer on the diffusion of Cu atoms from the Al matrix into the Ω-phase, the thickening resistance of the Ω-phase is significantly increased. Consequently, the Ω-phase exhibits enhanced thermal stability. The entire Ω-phase plate maintains an almost uniform thickness. While the length of the Ω-phase is prone to increase, its thickening is hindered. Therefore, after formation, the Ω-phase grows laterally along its primary growth direction.

Statistical analysis of the area fraction and average dimensions (length and width) of the Ω-phase precipitates was performed on dozens of TEM images taken from the Al–4.0Cu–0.5Mg–0.5Zn–0.5Mn–0.4Ag–(x = 0, 0.08, 0.16 and 0.24)Zr (in wt.%) alloys. The results show that the Al–4.0Cu–0.5Mg–0.5Zn–0.5Mn–0.4Ag alloy (x = 0) has the smallest Ω-phase area fraction of 8.43%. However, the Ω-phase precipitates within its matrix are the largest in both length and width compared to the other three Zr–containing alloys. Conversely, the Al–4.0Cu–0.5Mg–0.4Ag–0.16Zr alloy possesses the highest Ω-phase area fraction, while its Ω-phase precipitates exhibit the smallest average length and width.

It is evident that the Ω-phase area fraction and precipitate size (both length and width) exhibit a trend of initially decreasing and then increasing with the increasing Zr content in the alloy. Combining these findings with the prior EBSD analysis results, it is understood that the addition of Zr promotes the retention of more sub-grains and deformed structures within the matrix of the Al–4.0Cu–0.5Mg–0.5Zn–0.5Mn–0.4Ag–xZr (in wt.%) alloys, resulting in a higher dislocation density. This elevated dislocation density enhances the nucleation rate of the Ω-phase, thereby refining its size. Dai et al. [29] modified 2024 aluminum alloy by introducing Sc and Zr, and reported that the addition of minor amounts of Sc and Zr led to the formation of nanoscale Al_3_(Sc,Zr) particles. These particles served as preferential nucleation sites for the early precipitation and subsequent growth of the S-phase, thereby accelerating S-phase precipitation. In addition, they induced a particle-strengthening mechanism of Al_3_(Sc,Zr) after peak aging, which ultimately optimized the mechanical properties of the alloy. This trend is consistent with our findings.

**Figure 9:**
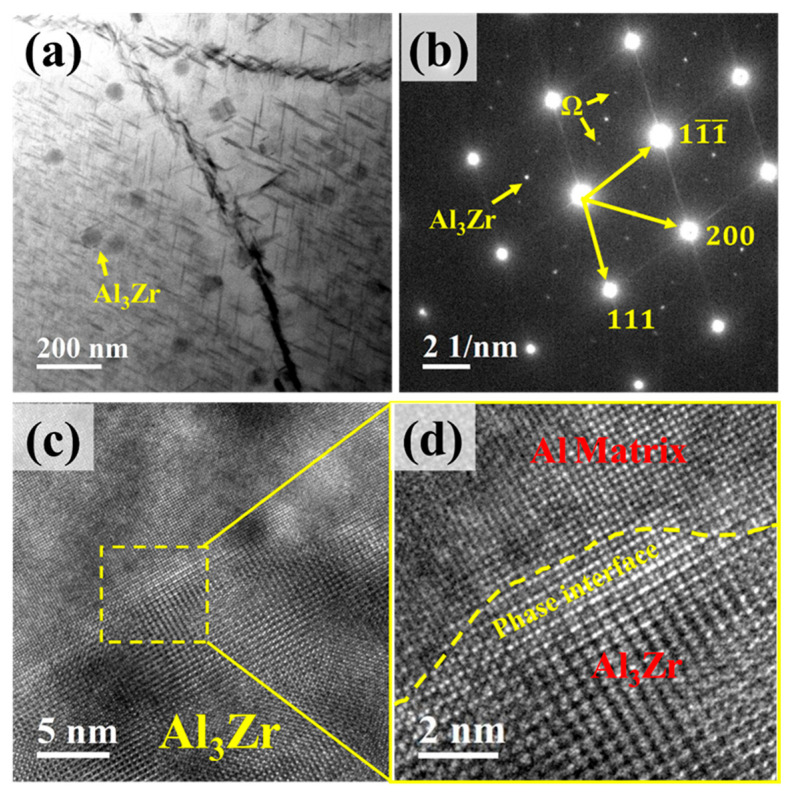
TEM micrographs of Al_3_Zr phase. (**a**) Distribution of Al_3_Zr phase; (**b**) Selected area electron diffraction (SAED) pattern corresponding to (**a**), incident beam direction // <011>α; (**c**) High-resolution TEM (HRTEM) image of Al_3_Zr phase; (**d**) Enlarged view of the boxed region in (**c**).

Figure 9a shows the TEM morphology of the Al_3_Zr phase within the alloy. It can be observed that the L1_2_–Al_3_Zr phase particles are dispersively distributed and are nanosized. Figure 9b presents the corresponding selected area diffraction pattern (SADP), where spots from both the Al_3_Zr phase and the Ω-phase are clearly visible. Figure 9c shows a high-resolution TEM (HRTEM) image of an Al_3_Zr precipitate, and Figure 9d displays its corresponding Fast Fourier Transform (FFT) pattern. The interface between the Al_3_Zr precipitate and the Al matrix is distinct.

**Figure 10:**
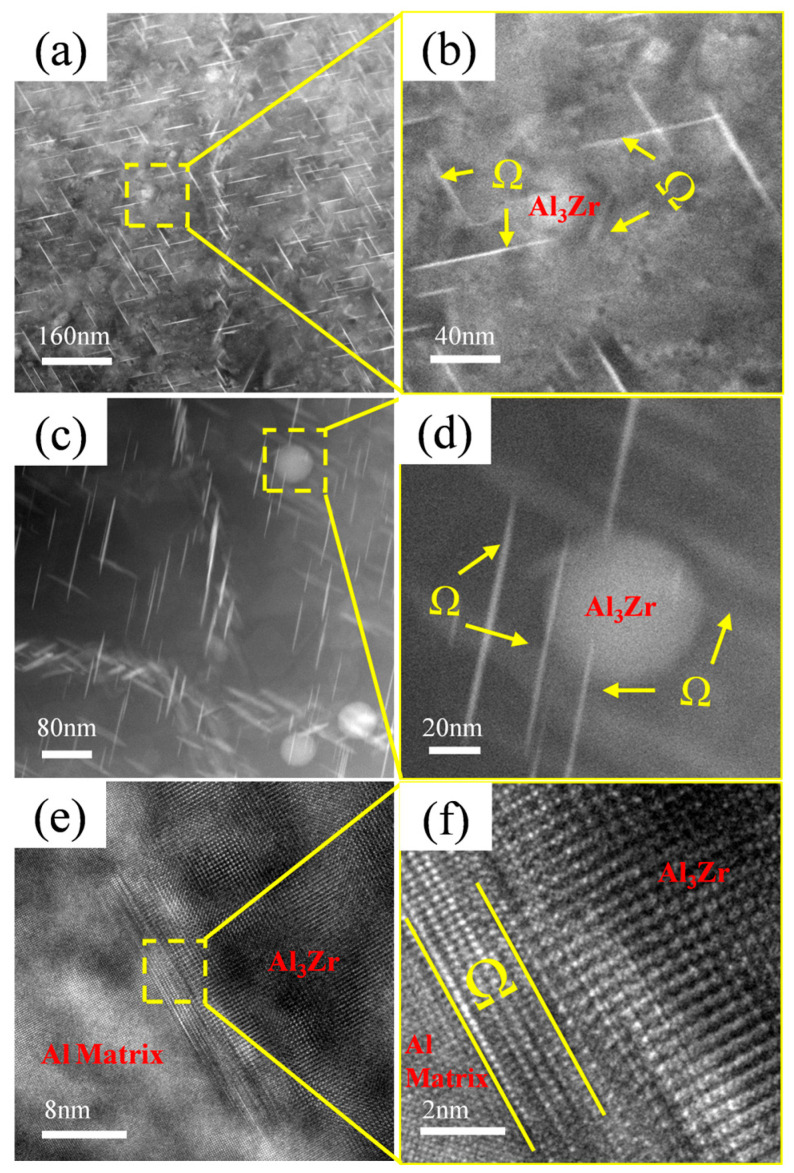
Ω-phase growing adjacent to Al_3_Zr phase. (**a**) Precipitate distribution in Al–4.0Cu–0.5Mg–0.5Zn–0.5Mn–0.4Ag–0.16Zr alloy; (**b**) Enlarged view of boxed region in (**a**); (**c**) Enlarged view of precipitate distribution; (**d**) Enlarged view of boxed region in (**c**); (**e**) HRTEM image of Al_3_Zr phase and Ω-phase; (**f**) Enlarged view of boxed region in (**e**).

During the study of precipitates, it was observed that the Ω-phase in the alloy tends to form adjacent to or in the vicinity of Al_3_Zr particles, as shown in Figure 10. Figure 10a,c reveal a high density of needle-like Ω-phase precipitates distributed near the Al_3_Zr particles in the alloy, with most of them in contact with the Al_3_Zr phase. Figure 10b,d shows magnified views of the boxed regions. Figure 10e displays the morphology of a small, incipient Ω-phase precipitate nucleating on an Al_3_Zr particle, and Figure 10f provides a magnified view of the boxed area in Figure 10e, clearly showing the narrow, nascent Ω-phase attached to the Al_3_Zr particle. Therefore, it can be inferred that, during Ω-phase formation, the pre-existing Al_3_Zr particles or the strain fields surrounding them can act as heterogeneous nucleation sites for the Ω-phase. This promotes heterogeneous nucleation of the Ω-phase, thereby increasing its nucleation rate and refining the Ω-phase precipitates in the alloy.

In the Al–4.0Cu–0.5Mg–0.5Zn–0.5Mn–0.4Ag–0.16Zr alloy, a higher number of finely dispersed, nanosized Al_3_Zr particles are formed. These particles effectively serve as heterogeneous nucleation sites for the Ω-phase. Consequently, this alloy exhibits the finest Ω-phase precipitates along with the highest Ω-phase area fraction. However, when the Zr content is excessively high (e.g., 0.24Zr), the Al_3_Zr particles undergo coarsening, and their population density decreases. This results in a diminished effectiveness of the Al_3_Zr particles as heterogeneous nucleation sites for the Ω-phase.

The mechanical properties of the peak-aged Al–4.0Cu–0.5Mg–0.5Zn–0.5Mn–0.4Ag–(x = 0, 0.08, 0.16, and 0.24)Zr (in wt.%) alloys are summarized in Table 2. With increasing Zr content, the trends in hardness, tensile strength, and yield strength are not simply linear; instead, these properties increase initially and then decrease. The Al–4.0Cu–0.5Mg–0.5Zn–0.5Mn–0.4Ag–0.16Zr alloy exhibits the highest values for hardness, tensile strength, and yield strength. However, the mechanical properties of the Al–4.0Cu–0.5Mg–0.5Zn–0.5Mn–0.4Ag–0.24Zr alloy slightly decrease with further Zr addition. Furthermore, the addition of Zr generally reduces the elongation to failure (ductility) of the alloys. Compared with conventional 2618–T6 alloy, the Al–4.0Cu–0.5Mg–0.4Ag–0.16Zr alloy exhibits an increase of 21.4 MPa in yield strength and 22.9 MPa in ultimate tensile strength, while the elongation is slightly reduced by 0.2% [30,31]. Overall, the designed alloy demonstrates superior room-temperature properties relative to 2618. Furthermore, the addition of Zr generates Al_3_Zr precipitates in the Al matrix, contributing to Orowan strengthening. However, excessive hard precipitates force dislocations to bypass them, increasing the resistance to dislocation motion. Under external stress, this results in local stress concentration around the precipitates, which acts as a crack–initiation site, facilitating microcrack formation and propagation and thereby reducing ductility.

**Figure 11:**
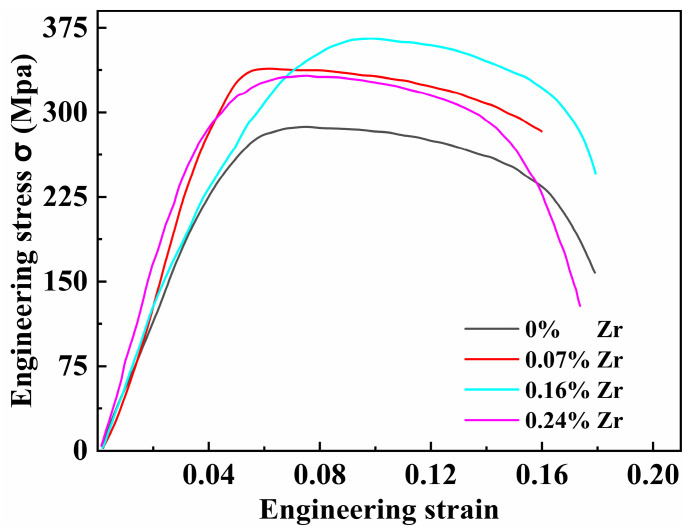
Tensile stress-strain curves of four alloys tested at 200 °C.

To investigate the effect of Zr content on high-temperature performance, tensile tests were conducted at 200 °C on all four peak-aged alloys. The results are presented in Figure 11 and Table 3. Compared to room temperature tensile properties, the mechanical properties of the Al–4.0Cu–0.5Mg–0.5Zn–0.5Mn–0.4Ag–(x = 0, 0.08, 0.16, and 0.24)Zr alloys at 200 °C show significant changes. The Al–4.0Cu–0.5Mg–0.5Zn–0.5Mn–0.4Ag alloy (x = 0) still maintains the highest elongation to failure. With increasing Zr content, the high-temperature yield strength gradually increases. The trend in high-temperature tensile strength resembles that observed at room temperature, with the Al–4.0Cu–0.5Mg–0.5Zn–0.5Mn–0.4Ag–0.16Zr alloy exhibiting the highest tensile strength.

Table 3 compares the tensile strength after high-temperature testing (200 °C) with the room temperature tensile strength for the alloys. After holding at 200 °C for 30 min, the Al–4.0Cu–0.5Mg–0.5Zn–0.5Mn–0.4Ag–0.16Zr alloy retains the highest tensile strength retention ratio of 0.798 (i.e., 79.8%). In contrast, the Al–4.0Cu–0.5Mg–0.5Zn–0.5Mn–0.4Ag alloy (x = 0) exhibits the poorest high-temperature resistance. The tensile strength retention of conventional 2618–T6 alloy is 56.2%, whereas the Al–4.0Cu–0.5Mg–0.4Ag–0.16Zr alloy shows a 41.2% higher retention compared with 2618–T6.

This study reveals the dual-function mechanism of Zr microalloying in Al–Cu–Mg–Ag alloys, with its core contradiction lying in the balance between grain refinement/recrystallization suppression and microstructural homogeneity. At 0.16 wt.% Zr content, Al_3_Zr particles achieve optimal synergy in size (~20 nm) and distribution density. Nano-scale Al_3_Zr phases effectively suppress recrystallization through Zener pinning (recrystallization fraction reduced to 27.95%), with retained deformed structures providing high-density dislocations as nucleation sites for Ω-phase. Secondly, acting as heterogeneous nucleation substrates, they significantly refine Ω-phase precipitates (smallest average size) and narrow precipitate-free zone (PFZ) widths (Figure 10).

This coupled mechanism of “dislocation density enhancement + heterogeneous nucleation synergy” enables peak strength achievement (461.9 MPa at RT, 368.8 MPa at 200 °C). However, excessive Zr (0.24 wt.%) induces multi–scale inhomogeneity. Coarsened Al_3_Zr particles (>50 nm) increase Schmid factor distribution dispersion (Figure 6h), degrading deformation compatibility. Drastically reduced recrystallization (19.91%) with elevated sub-grain fraction (34.32%) transforms coarse deformed grains (red regions, Figure 5n) into stress concentration sources. Bimodal grain size distribution (Figure 5p) promotes premature intergranular crack initiation (Figure 6h).

Notably, the high-temperature performance degradation (strength retention: 0.750 for 0.24Zr vs. 0.798 for 0.16Zr) primarily stems from PFZ thermal stability differences. According to the Ω-phase growth mechanism (Figure 8f), Al_3_Zr particles inhibit Cu atom diffusion by adsorbing Ag–Mg co-clusters. Coarsened Al_3_Zr particles diminish this effect due to reduced specific surface area, accelerating PFZ widening at high temperatures. This phenomenon aligns with the “solute trapping model” proposed by Murayama et al. [32], where interfacial energy barriers of nanoparticles are key to stabilizing PFZs. From an engineering perspective, the Al–4.0Cu–0.5Mg–0.5Zn–0.5Mn–0.4Ag–0.16Zr alloy’s 79.8% high-temperature strength retention surpasses commercial 2618A alloy (~70% at 150 °C/100 h). However, its plasticity (δ = 15.2%) remains constrained by brittle fracture tendencies induced by high Ω-phase density. Future optimization could involve composite microalloying, forming Al_3_(Sc,Zr) core-shell structures to enhance pinning efficiency or strain aging utilizing pre-deformation dislocation networks to refine Ω-phase distribution [33].

## 4. Conclusions

This study systematically investigates the influence of heat treatment parameters and Zr content (0–0.24 wt.%) on the mechanical properties and precipitate evolution of Al–4.0Cu–0.5Mg–0.5Zn–0.5Mn–0.4Ag–xZr alloys. Key findings are summarized as follows:

(i) The Al–4.0Cu–0.5Mg–0.5Zn–0.5Mn–0.4Ag–0.16Zr alloy demonstrates superior mechanical properties at both room and elevated temperatures compared to other compositions, achieving a peak tensile strength of 368.8 MPa and retaining the highest tensile strength retention ratio of 0.798 at 200 °C.
(ii) The introduction of Zr facilitates the precipitation of Al_3_Zr phase, effectively pinning grain boundaries and dislocations to enhance recrystallization resistance while preserving deformed microstructures. However, excessive Zr content induces non-uniform grain size distribution and significant Schmid factor variations among grains. This heterogeneity promotes stress concentration near coarse deformed grains with high Schmid factors, triggering dislocation accumulation that initiates premature intergranular cracking and ultimately degrades alloy performance.
(iii) Al_3_Zr particles serve as heterogeneous nucleation sites for Ω-phase precipitation. When dispersively distributed near grain boundaries, they accelerate Ω-phase nucleation at these interfaces, refine grain boundary precipitates, and narrow precipitate-free zone (PFZ) widths. These coordinated microstructural modifications collectively enhance the alloy’s mechanical properties.

## Figures and Tables

**Table 1:** The actual composition of the aluminum alloy used in the experiment (wt.%).

	Cu	Mg	Ag	Zn	Mn	Zr	Al
Alloy 1	4.06	0.47	0.41	0.44	0.5	0	Balance
Alloy 2	4.24	0.41	0.39	0.49	0.49	0.07	
Alloy 3	4.31	0.5	0.34	0.42	0.53	0.15	
Alloy 4	4.03	0.46	0.42	0.51	0.52	0.24	

**Table 2:** Mechanical properties in the peak-aged condition of Al–4.0Cu–0.5Mg–0.5Zn–0.5Mn–0.4Ag–(x = 0, 0.08, 0.16 and 0.24)Zr (in wt.%) alloys and 2618A alloys.

Alloys	Hardness (HV)	Tensile Strength (MPa)	Yield Strength (MPa)	Elongation (%)
Al–4.0Cu–0.5Mg–0.5Zn–0.5Mn–0.4Ag	141.81	439.8	356.9	16.1
Al–4.0Cu–0.5Mg–0.5Zn–0.5Mn–0.4Ag –0.08Zr	145.67	448.7	377.4	15.1
Al–4.0Cu–0.5Mg–0.5Zn–0.5Mn–0.4Ag–0.16Zr	152.24	461.9	390.4	15.2
Al–4.0Cu–0.5Mg–0.5Zn–0.5Mn–0.4Ag–0.24Zr	151.4	453.6	375.2	14.9
2618A		441	372	10

**Table 3:** Mechanical properties in the peak-aged condition of Al–4.0Cu–0.5Mg–0.5Zn–0.5Mn–0.4Ag–(x = 0, 0.08, 0.16, and 0.24)Zr (in wt.%) alloys and 2618A at 200 °C.

Alloys	Tensile Strength (MPa)	Tensile Strength (MPa)	Elongation (%)	Strength Retention (%)
Al–4.0Cu–0.5Mg–0.5Zn–0.5Mn–0.4Ag	285.87	279.42	19.5	0.650
Al–4.0Cu–0.5Mg–0.5Zn–0.5Mn–0.4Ag–0.08Zr	337.68	333.37	17.4	0.753
Al–4.0Cu–0.5Mg–0.5Zn–0.5Mn–0.4Ag–0.16Zr	368.8	346.71	18.1	0.798
Al–4.0Cu–0.5Mg–0.5Zn–0.5Mn–0.4Ag–0.24Zr	340.4	337.38	16.9	0.750
2618A	230	203	22.4	0.562

## Data Availability

The original contributions presented in this study are included in the article. Further inquiries can be directed at the corresponding author.

## Abbreviations

The following abbreviations are used in this manuscript:

PFZs	Precipitate-free zones
OM	Optical microscopy
SEM	Scanning electron microscopy
EBSD	Electron backscatter diffraction
TSL	Tex Sem Lab
IPF	Inverse pole figure
TEM	Transmission electron microscopy
BSE	Backscattered electron
HABs	High-Angle Boundaries
LABs	Low-Angle Boundaries
FFT	Fast Fourier Transform
